# Proteomic profiling of sudden cardiac death with acquired cardiac hypertrophy

**DOI:** 10.1007/s00414-023-03038-6

**Published:** 2023-06-07

**Authors:** Yu Kakimoto, Atsushi Ueda, Masatoshi Ito, Masayuki Tanaka, Tomoko Kubota, Shotaro Isozaki, Motoki Osawa

**Affiliations:** 1 Department of Forensic Medicine, Tokai University School of Medicine, Kanagawa, Japan; 2 Support Center for Medical Research and Education, Tokai University, Kanagawa, Japan

**Keywords:** Sudden cardiac death, Cardiac hypertrophy, Fibrosis, Proteomics, Sarcomere proteins, MYH7, MYL3

## Abstract

**Background:**

Cardiac hypertrophy, which develops in middle-aged and older individuals as a consequence of hypertension and obesity, is an established risk factor for sudden cardiac death (SCD). However, it is sometimes difficult to differentiate SCD with acquired cardiac hypertrophy (SCH) from compensated cardiac hypertrophy (CCH), at autopsy. We aimed to elucidate the proteomic alteration in SCH, which can be a guideline for future postmortem diagnosis.

**Methods:**

Cardiac tissues were sampled at autopsy. SCH group consisted of ischemic heart failure, hypertensive heart failure, and aortic stenosis. CCH group included cases of non-cardiac death with cardiac hypertrophy. The control group comprised cases of non-cardiac death without cardiac hypertrophy. All patients were aged > 40 years, and hypertrophic cardiomyopathy was not included in this study. We performed histological examination and shotgun proteomic analysis, followed by quantitative polymerase chain reaction analysis.

**Results:**

Significant obesity and myocardial hypertrophy, and mild myocardial fibrosis were comparable in SCH and CCH cases compared to control cases. The proteomic profile of SCH cases was distinguishable from those of CCH and control cases, and many sarcomere proteins were increased in SCH cases. Especially, the protein and mRNA levels of MYH7 and MYL3 were significantly increased in SCH cases.

**Conclusion:**

This is the first report of cardiac proteomic analysis in SCH and CCH cases. The stepwise upregulation of sarcomere proteins may increase the risk for SCD in acquired cardiac hypertrophy before cardiac fibrosis progresses significantly. These findings can possibly aid in the postmortem diagnosis of SCH in middle-aged and older individuals.

**Supplementary Information:**

The online version contains supplementary material available at 10.1007/s00414-023-03038-6.

## Introduction

Sudden cardiac death (SCD) refers to unexpected death not attributable to an extracardiac cause, occurring usually within 1 h of symptom onset (or within 24 h of last being seen in good health if the death is unwitnessed) [1]. Lethal arrhythmia due to coronary artery disease is considered the major cause of SCD in middle-aged and older individuals [2, 3]. High levels of coronary atherosclerotic plaques are often observed at autopsy in middle-aged and older individuals who die of non-cardiac cause. Thus, together with coronary plaque, myocardial molecular remodeling can also be a substrate for lethal arrythmia which can contribute to SCD in the middle-aged and older population.

Cardiac hypertrophy is an established risk factor for SCD, known to increase the risk of SCD with an odds ratio of 2.5–4.2 [4–6]. Among patients with cardiac hypertrophy, hypertrophic cardiomyopathy (HCM) has been cited as a leading cause of SCD in younger populations, whereas hypertension/obesity-related cardiac hypertrophy is a more common finding in SCD in middle-aged and older populations [6–8]. Physiologically, cardiac hypertrophy occurs in response to pressure and volume overload, progressing through the compensated phase without symptoms until heart failure in the decompensated phase [9, 10]. Although years or decades may pass between the first episode of heart failure and cardiac death in the decompensated phase, some patients die suddenly before symptoms of heart failure appear. Given accelerated population aging in recent years, the clinical impact of SCD with acquired cardiac hypertrophy (SCH) remains substantial and is likely to increase.

At autopsy, it is sometimes difficult to grossly differentiate SCH from compensated cardiac hypertrophy (CCH), which does not contribute to the cause of death in middle-aged and older generations. Histological findings such as coronary atherosclerosis and cardiac hypertrophy are not sufficient to diagnose SCD; circumstantial information of sudden death and negative results of toxic screening are often necessary for the final diagnosis of SCD. Therefore, further information of cardiac molecular alterations that contribute to SCD is essential to differentiate SCH from CCH cases at postmortem examination.

Recently, genetic variants have gained attention as an explanation of the variable risk of SCD in patients with acquired cardiac diseases. Postmortem genetic testing has revealed that 3–10% of SCH cases are likely caused by pathogenic variants, where HCM is histologically denied [8, 11–13]. These results indicate that commonly acquired cases of cardiac hypertrophy may interact with genetic variants. However, the broad genetic panel mostly detects variants of unknown significance; therefore, genetic screening confers less diagnostic utility in cases of acquired cardiac hypertrophy than in cases of inherited cardiomyopathy, as it cannot further evaluate the pathogenicity of variants.

In this study, we aimed to elucidate the proteomic changes in middle-aged and older human heart, which can directly contribute to the pathology of SCH and may be used for the postmortem diagnosis.

## Materials and methods

### Study design

Forensic cases in which patients were discovered within 24 h after death were enrolled in this study (Table 1 and Online Resource 1). All patients were > 40 years of age. To focus on the acquired cardiac hypertrophy, HCM cases were not included. As well, to focus on the early proteomic changes, cases after cardiac operation or grossly presenting cardiac fibrosis were not included. This study was approved by the institutional ethical committee (21R177), and informed consent was obtained from the relatives of all patients.

**Table 1:** Clinical characteristics of included patients

	SCH (*n* = 10)	CCH (*n* = 10)	Control (*n* = 10)
Cause of death	SCD: IHF (6), HHF (3), AS (1)	Accident (8), Cerebral hemorrhage (2)	Accident (8), Pneumonia (2)
Age, years	63 (41–82)	59 (43–74)	58 (42–76)
Sex	10 men	10 men	8 men, 2 women
BMI (kg/m^2^)	25.0 (17.5–34.5) *	25.4 (19.4–28.4) *	20.6 (14.0–27.5)
BNP (pg/mL)	30.0 (2.0–56.6)	16.4 (2.0–81.5)	6.0 (2.0–21.8)

*SCD* sudden cardiac death; *SCH* SCD with acquired cardiac hypertrophy; *CCH* compensated cardiac hypertrophy; *IHF* ischemic heart failure; *HHF* hypertensive heart failure; *AS* aortic stenosis; *BMI* body mass index; *BNP* B-type natriuretic peptide whose lowest detectable level is 2.0 pg/mL in our assay. * *p* < 0.05; ** *p* < 0.001, compared with control cases. Individual characteristics are detailed in Online Resource 1

SCD was defined as death within 1 h of symptom onset or unwitnessed death with the individual having normal health within 24 h before death [1]. Cardiac hypertrophy was diagnosed as heart weight/body height (g/cm) > 2.38 in men and > 2.15 in women, respectively [6]. SCH was defined as SCD with acquired cardiac hypertrophy, consisting of ischemic heart failure, hypertensive heart failure, and aortic stenosis. Ischemic heart failure was diagnosed based on a high level of coronary atherosclerosis or coronary thrombus, and exclusion of other causes of death. Hypertensive heart failure was diagnosed based on a clinical history of hypertension and hypertension-related changes such as nephrosclerosis in those without a high level of coronary atherosclerotic plaques. Because there are some cases in which cardiac hypertrophy contributes relatively less to the cause of death, we set CCH as the subcontrol group in this study. The CCH group included cases of non-cardiac death in patients with cardiac hypertrophy without a clinical history of heart failure. The control group included cases of non-cardiac death in patients without cardiac hypertrophy or a clinical history of heart failure.

### Tissue sampling and histological analysis

The body was stored at 4 ℃ until autopsy, and the cardiac tissue was extracted during autopsy. At the middle level between the base and apex, small tissue samples from the middle layer of the left ventricular free wall were removed and immediately frozen at -80 ℃ and stored until protein isolation. The other cardiac tissues were preserved in 10% formalin, and 4-μm sections were stained with hematoxylin and eosin (H&E), and picrosirius red dyes. Microscopic measurements were performed in the sections from the tissues obtained from the middle layer of the left ventricular free wall. The endocardium, epicardium, and trabeculae carneae were excluded. The cardiomyocyte diameter was measured at the nuclear point with H&E staining at × 200 magnification, and the values were averaged from 10 fields per case. The myocardial fibrosis was quantitatively assessed with picrosirius red at × 40 magnification using Image J software (https://imagej.net/), and the percentage to the total image were averaged in 10 fields per case [14, 15].

### Proteomic analysis

Cardiac tissue samples were homogenized using a Shake Master Neo system (Bio Medical Science, Tokyo, Japan) in an equal volume of phase-transfer surfactant-based lysis buffer containing 12 mM deoxycholic acid, 12 mM sodium N-lauroylsarcosine, 50 mM ammonium bicarbonate, and protease inhibitors [16]. After centrifugation at 15,000 × *g* for 5 min, the collected supernatant was subjected to protein quantification using a DC protein assay (Bio-Rad, Hercules, CA, USA).

For quantitative proteomic analysis, 5 μg protein from the supernatant samples were used. Protein samples were enzymatically digested with mass spectrometry (MS)-grade trypsin (Promega, Madison, WI, USA). Digested samples were dried using a SpeedVac vacuum concentrator (Thermo Fisher Scientific, Waltham, MA, USA) and dissolved in 50 mM ammonium bicarbonate buffer containing 0.1% trifluoroacetic acid (TFA). Samples were loaded onto C18 StageTips [17] and subsequently eluted with 30 and 60% acetonitrile in 50 mM ammonium bicarbonate. The eluted samples were dried using a SpeedVac vacuum concentrator and dissolved in 13 μL of 2% acetonitrile in 0.1% formic acid, of which 6 μL was applied for liquid chromatography–MS (LC–MS). LC–MS-grade ultrapure water and acetonitrile were purchased from Merck (Darmstadt, Germany). TFA was purchased from Wako Pure Chemical Industries (Osaka, Japan). LC–MS grade formic acid was obtained from Thermo Fischer Scientific.

Quantitative proteomic analysis was performed as previously described [18], with minor modifications. A Q-Exactive mass spectrometer coupled with an UltiMate 3000 High Performance Liquid Chromatography (HPLC) system (Thermo Fisher Scientific) was used. The injected peptides were separated using a nano-LC capillary column (75 μm × 180 mm, C18, 3 μm; Nikkyo Technos, Tokyo, Japan) at a flow rate of 300 nL/min. The aqueous mobile phase (eluent A) was 0.1% formic acid in 2% acetonitrile and the organic mobile phase (eluent B) was 0.1% formic acid in 95% ACN. The elution gradient was 2–33% B for 120 min. Data were acquired using a data-dependent acquisition protocol in which an MS1 scan ranging from 350 to 1,500 m*/z* was selected, followed by higher-energy C-trap dissociation (HCD)-based MS/MS fragmentation against the 20 highest precursor peaks. Raw data files were subjected to protein identification analysis using Mascot server ver.2.7 (Matrix Science, London, UK). Protein searches were conducted against the SwissProt human database (January 2020). The mass tolerances for the precursor and product ions were 5 ppm and 0.05 Da, respectively. Two trypsin missed cleavages and variable modifications of N-terminal acetylation, N-terminal carbamylation, cysteine carbamidomethylation, and methionine oxidation were allowed. Proteins were identified with FDR 1% and peptide ion score > 30.

Label-free quantitative analysis of the identified proteins was performed using the Progenesis QI for proteomics v2 software (Nonlinear, Durham, NC, USA). Protein levels were normalized using the ratiometric method in log space, and a *p*-value < 0.05 in an analysis of variance among the groups was considered significant. Principal component analysis and differential analysis were performed using SIMCA (Infocom, Tokyo, Japan). Gene ontology analysis was performed using DAVID Bioinformatics Resources 6.8 (https://david.ncifcrf.gov/), and a *p*-value < 0.05 obtained using the Benjamini–Hochberg method was considered significant.

### Quantitative polymerase chain reaction (qPCR)

Total RNA was isolated from the cardiac tissues using TRIzol reagent (Invitrogen, Carlsbad, CA, USA) and reverse-transcribed into cDNA using a High-Capacity cDNA Reverse Transcription Kit (Applied Biosystems, Waltham, MA, USA). qPCR was performed using a StepOnePlus Real-Time PCR system (Applied Biosystems). Triplicate C*t* values obtained for tenfold diluted cDNA were averaged, and the relative expression of target mRNA was determined via the ΔΔC*t* method [19], using GAPDH as an internal control. The relative levels were normalized to the average values of the control cases. The primers for MYH7 (Hs01110632_m1), MYL3 (Hs00264820_m1), ACTC1(Hs01109515_m1), and GAPDH (4333764F) were obtained using TaqMan Assay (Applied Biosystems).

### Statistical analysis

Multiple comparisons were performed using the Steel–Dwass test for clinical value, qPCR, and histological results in Excel Statistics 2015 (SSRI, Tokyo, Japan). Differences with *p* values < 0.05 were considered statistically significant.

## Results

### Histological analysis

**Fig. 1:**
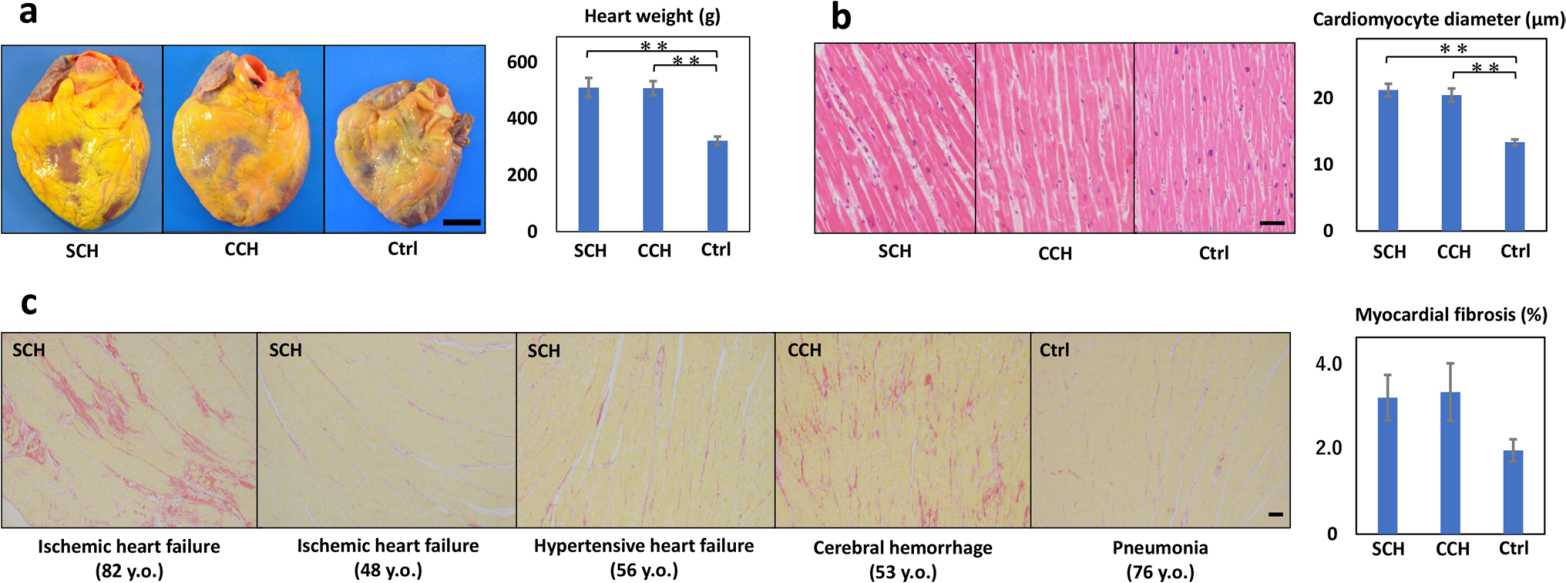
Histological characteristics of the specimens obtained from the cases. **a** Representative macroscopic images of the heart. There was comparable increase in heart weights in SCH and CCH cases when compared with that in control cases. Scale bar = 3 cm. **b** Representative microscopic images of cardiomyocytes with hematoxylin and eosin staining. The cardiomyocytes diameters are increased in equal proportions in SCH and CCH cases when compared with that in control cases. Scale bar = 50 μm. **c** Representative microscopic images with picrosirius red staining. The myocardial fibrosis fractions are moderately visible in SCH and CCH cases when compared with that in control cases. Scale bar = 200 μm. Graphical error bars represent mean ± SE. *p < 0.05, **p < 0.001. SCH, sudden cardiac death with acquired cardiac hypertrophy; CCH, compensated cardiac hypertrophy

The heart weights of SCH (511 ± 34 g) and CCH (508 ± 25 g) were significantly heavier than the control cases (322 ± 15 g), but there was no significant difference between SCH and CCH cases (Fig. 1a). As well, the cardiomyocyte diameter was significantly increased in SCH (21.2 ± 0.9 µm) and CCH (20.5 ± 1.0 µm) compared with the control cases (13.4 ± 0.4 µm), but there was no significant difference between SCH and CCH cases (Fig. 1b). No significant accumulation of inflammatory cells was observed in any case.

The myocardial fibrosis level was moderately increased in SCH (3.1 ± 0.5%) and CCH (3.2 ± 0.7%) compared with the control cases (1.9 ± 0.3%), but no significant substantial variation was observed (Fig. 1c). Elderly SCH cases with ischemic heart failure or aortic stenosis presented the most prominent patchy fibrosis, while the middle-aged SCH cases presented a little to moderate levels of myocardial fibrosis. In contrast, CCH cases with cerebral hemorrhage presented more prominent diffuse fibrosis than SCH cases with hypertensive heart failure. In the control cases, a little to moderate levels of myocardial fibrosis was observed elderly cases.

### Proteomic analysis

Proteomic profiles were obtained from three patients with SCH (all hypertensive heart failure), three with CCH, and four control patients. A total of 9,581 peptides and 1,551 proteins were identified, and the abundance of 140 proteins significantly differed between the groups (Online Resources 2 and 3). Among the top 1,000 abundant proteins, 19 exhibited significant differences with fold changes > 5 among the groups, and most of them were upregulated in the SCH group (Table 2).

**Table 2:** Major cardiac proteins differentially expressed based on proteomic analysis

Gene ID	Description	*p*-value	Fold change	Highest group	Lowest group	Average abundance		
						A: SCH	B: CCH	C: Control
MYH7	Myosin-7 (β-Myosin heavy chain)	0.00001	31.7	A	C	4,398,718,463	240,223,132	138,631,218
MYL3	Myosin light chain 3	0.00515	5.4	A	C	2,278,403,475	774,826,120	419,683,634
ACTC1	Actin, alpha cardiac muscle 1	0.00011	6.4	A	C	661,847,239	111,049,697	102,836,296
ACTA1	Actin, alpha skeletal muscle	0.01325	5.0	A	C	237,838,909	79,671,590	47,534,401
H4C1	Histone H4	0.02068	6.1	A	C	126,050,127	63,734,082	20,503,322
BLVRA	Biliverdin reductase A	0.00001	7.4	A	B	11,870,823	1,594,859	1,906,043
RBP4	Retinol-binding protein 4	0.00050	6.1	A	C	6,842,534	2,062,599	1,124,422
MYH9	Myosin-9	0.00014	46.4	A	B	5,151,286	111,106	130,214
MYOM3	Myomesin-3	0.00319	6.9	A	C	4,905,559	2,122,198	714,203
MT-CO1	Cytochrome c oxidase subunit 1	0.00390	6.4	A	C	3,188,028	1,204,247	501,341
MT-ATP6	ATP synthase subunit a	0.00442	5.4	C	A	2,604,003	11,058,033	14,132,003
PSMA5	Proteasome subunit alpha type-5	0.00006	9.1	A	C	1,780,039	355,736	196,202
MACROH2A1	Core histone macro-H2A.1	0.04699	5.2	A	C	1,695,102	813,034	328,393
MYH11	Myosin-11	0.00118	33.0	A	C	1,229,860	43,050	37,230
C1QB	Complement C1q subcomponent subunit B	0.02465	5.5	A	B	1,165,830	210,848	535,773
PSMB1	Proteasome subunit beta type-1	0.00014	13.7	A	C	1,072,370	194,629	78,070
RHOC	Rho-related GTP-binding protein RhoC	0.03362	8.4	C	A	232,937	834,397	1,948,366
SLFN12L	Schlafen family member 12-like	0.01401	8.0	C	A	135,208	498,154	1,075,500
THBS4	Thrombospondin-4	0.00455	14.9	B	A	69,740	1,035,738	691,891

*ATP* adenosine triphosphate; *GTP* guanosine-5'-triphosphate; *SCD* sudden cardiac death; *SCH* SCD with acquired cardiac hypertrophy; *CCH* compensated cardiac hypertrophy

**Fig. 2:**
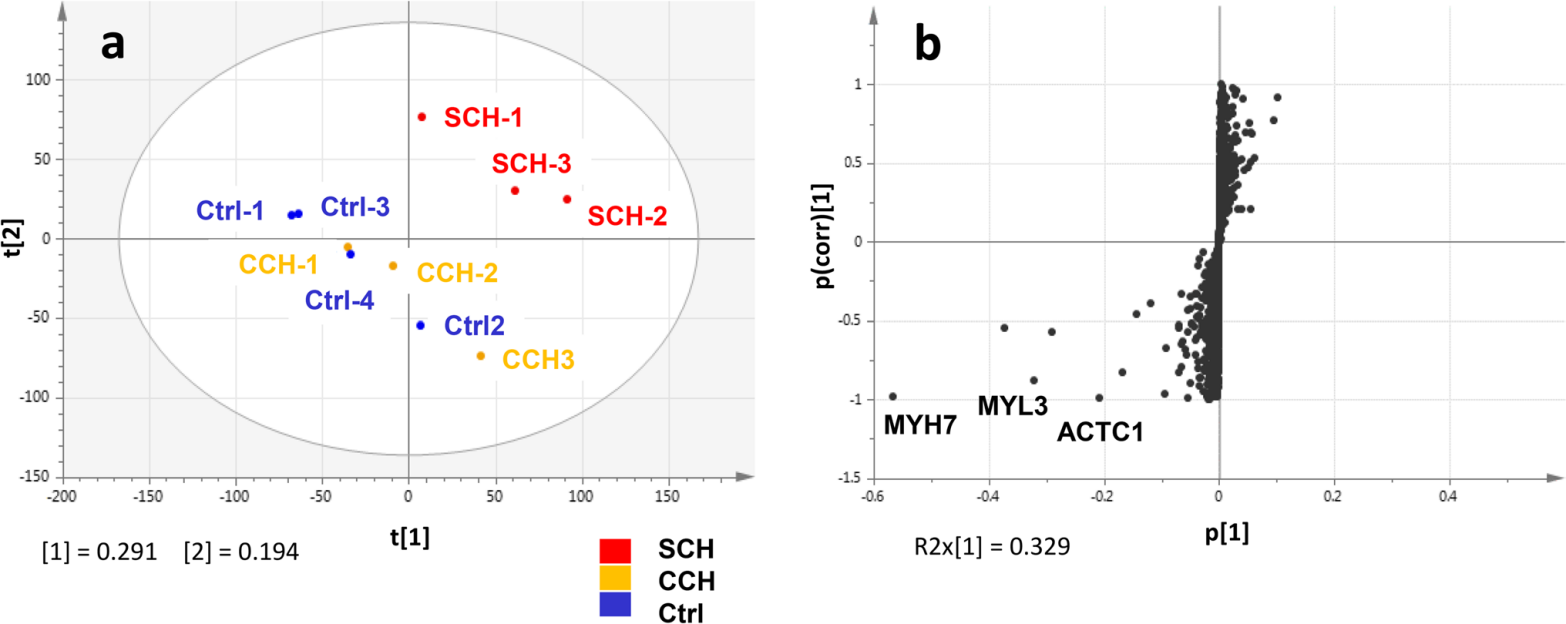
Multivariate analysis of the proteomic data. **a** Principal component analysis of total peptides. **b** S-plot loading diagram for the differentiation of the total proteins between SCH and CCH. SCH, sudden cardiac death with acquired cardiac hypertrophy; CCH, compensated cardiac hypertrophy

Principal component analysis of the total peptides indicated that SCH cases had profiles distinguishable from those of CCH and control cases, with the latter two exhibiting relatively similar profiles (Fig. 2a). Differential analysis of the total proteins revealed some sarcomeric proteins, including MYH7, MYL3, and ACTC1, that characterized SCH (vs. CCH) (Fig. 2b).

Gene ontology analysis of the 85 proteins that were significantly upregulated in SCH revealed their significant contributions to proteasomes, cardiac muscle contraction, and tight junction function (Table 3). Similarly, analysis of the 41 proteins that were significantly downregulated in SCH revealed their moderate contributions to oxidative phosphorylation and ribosome function (Table 4).

**Table 3:** Functional annotation summary of highly abundant proteins in SCH cases

Term	Count	%	Gene ID	*p*-value
Proteasome	11	10.9	PSMA4, PSMA5, PSMB4, PSMB5, PSMC1, PSMC4, PSMC5, PSMC6, PSMB1, PSMD1PS, MD12	6.30E-10
Cardiac muscle contraction	9	8.9	MYH7, ACTC1, MYL3, TPM1, TPM2, MT-CO1, MT-CO2, COX5A, COX7C	2.29E-05
Tight junction	9	8.9	MYH4, MYH3, MYH9, MYH11, MYH13, MYH14, MYH15, ACTB, MYL12B	4.81E-05
Ribosome	8	7.9	RPS2, RPS9, RPS27A, RPLP2, MRPS7, RPL17, RPL27, RPL34	0.00704
Adrenergic signaling in cardiomyocytes	7	6.9	MYH7, ACTC1, TPM1, TPM2, MYL3, PPP1CB, PPP1CC	0.03677

*SCH* sudden cardiac death with acquired cardiac hypertrophy

**Table 4:** Functional annotation summary of weakly abundant proteins in SCH cases

Term	Count	%	Gene ID	*p*-value
Oxidative phosphorylation	5	9.6	COX15, MT-ATP6, NDUFB1, NDUFV1, NDUFA5	0.034
Ribosome	5	9.6	RPS5, RPL15, RPLP1, MRPL23, MRPL27	0.034

*SCH* sudden cardiac death with acquired cardiac hypertrophy

### qPCR

**Fig. 3:**
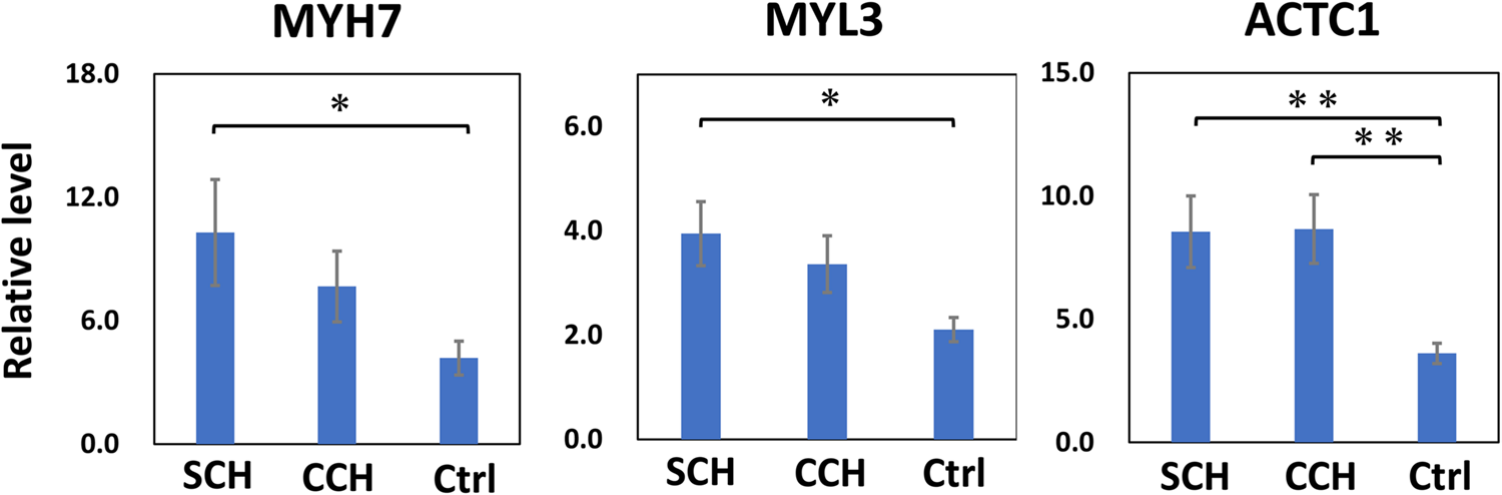
qPCR for MYH7, ACTC1, and MYL3. Quantitative PCR was performed using GAPDH as an endogenous control in human hearts. Error bars represent mean ± SE. *p < 0.05, **p < 0.001. SCH, sudden cardiac death with cardiac hypertrophy (*n* = 10); CCH, compensated cardiac hypertrophy (*n* = 10); Ctrl, control (*n* = 10)

Levels of MYH7 and MYL3 mRNA were significantly increased in SCH and moderately increased in CCH, when compared with levels observed in control cases (Fig. 3). The level of ACTC1 expression was also significantly higher in SCH and CCH cases than that in control cases.

## Discussion

Our study focused on acquired cardiac hypertrophy which accompanies obesity, hypertension, coronary arteriosclerosis, or aortic stenosis, which are common diseases of middle-aged and older adults. To the best of our knowledge, this study is the first to reveal proteomic changes in human cardiac tissue samples of patients with acquired cardiac hypertrophy resulting in SCD. Interestingly, protein and mRNA levels of MYH7, MYL3, and ACTC1, the major sarcomere genes whose variants can cause inherited cardiomyopathies such as HCM [20–22], and dilated cardiomyopathy [23, 24] were significantly increased in SCH cases. Studies have reported that some types of genetic mutations in these sarcomere proteins may increase the risk of SCD in patients with inherited cardiomyopathy [25–28]. The proteomic analysis in this study indicates that both qualitative and quantitative changes in these sarcomere proteins can influence cardiac function and increase the risk of SCD. Moreover, the proteomic changes here preceded the histological changes: the levels of sarcomere proteins were increased stepwise from CCH to SCH, while the levels of myocardial hypertrophy and fibrosis were unchanged. Thus, the elevation of sarcomere proteins may differentiate SCH from CCH in the early phase acquired cardiac hypertrophy before the myocardial fibrosis, a classical pathological finding of heart failure, progresses significantly.

The myosin heavy chain (MHC) is a functional myosin motor molecule, and two isoforms, α-MHC encoded by MYH6 and β-MHC encoded by MYH7, are expressed in the mammalian heart. The contractile velocity and energy consumption are two- to three-fold higher for α-MHC than those for β-MHC [29, 30]. In the rodent ventricle, with > 90% of the total MHC proteins, α-MHC predominates and contributes to rapid contraction [31, 32]. Meanwhile, β-MHC predominates with > 95% of the total MHC proteins in the human ventricle and results in a lower resting heart rate [33, 34]. Ventricular MYH6 significantly decreases and MYH7 significantly increases in the rodent heart with pressure or volume overload [34–36]. In contrast, studies suggest that ventricular MYH7 does not increase as significantly in human heart failure as in rodent models of heart failure [37–39], and decreased levels of MYH7 have been observed at the end of heart failure in some clinical studies [40, 41]. Thus, direct extrapolation from model animals, such as rats and mice, to humans is often challenging in cardiac research [42, 43]. Molecular analysis of human cardiac tissue samples can produce valuable data for understanding human cardiac pathology. Moreover, SCD samples can be collected and studied in detail in forensics rather than general clinical field, because of the accessibility to the SCD cases outside the hospital. Therefore, analyzing autopsy tissue samples to reveal the molecular pathology of SCD can be an important objective for the forensic pathologists.

This study has some practical limitations. First, the sample size was limited as we selected relatively fresh cases of autopsy. Although postmortem changes have often prevented the large-scale molecular studies to date, we believe that accrual of SCD cases in small-size studies can help to elucidate the pathology and contribute to accurate postmortem diagnosis in the future. Second, there were some discrepancies between the quantitative results obtained using proteomics and qPCR in this study, which may have been caused by the heterogeneous distribution of the target proteins in the heart. Moreover, gene ontology analysis revealed that altered proteasome activity in SCH may decrease protein clearance, resulting in the accumulation of sarcomere proteins without dramatic alterations in their mRNA levels. Future studies including microscopic sampling with microdissection and analyses at the single-cell level, may reveal the more precise pathology of SCH in the human heart.

## Conclusions

This is the first report of proteomic analysis in SCH and CCH using human cardiac tissue samples. Histologically SCH and CCH cases presented equal levels of significant myocardial hypertrophy and mild fibrosis. Meanwhile, the proteomic profiling demonstrated the significant upregulation of sarcomere proteins such as MYH7 and MYL3 in SCH cases. The stepwise upregulation of the sarcomere proteins from CCH to SCH may increase the risk for SCD in the acquired cardiac hypertrophy, and these findings can possibly be useful in the postmortem diagnosis of SCH in middle-aged and older individuals.

## Supplementary Information

Below is the link to the electronic supplementary material.

Supplementary file1 (XLSX 20 KB)

Supplementary file2 (XLSX 2415 KB)

Supplementary file3 (XLSX 367 KB)

## Data Availability

All data generated or analyzed during this study are included in this published article and its supplementary information files.
